# Systematic identification and characterization of repeat sequences in African swine fever virus genomes

**DOI:** 10.1186/s13567-022-01119-9

**Published:** 2022-12-02

**Authors:** Zhaozhong Zhu, Shengqiang Ge, Zena Cai, Yifan Wu, Congyu Lu, Zheng Zhang, Ping Fu, Longfei Mao, Xiaodong Wu, Yousong Peng

**Affiliations:** 1 Bioinformatics Center, College of Biology, Hunan Provincial Key Laboratory of Medical Virology, Hunan University, Changsha, 410082 China; 2 China Animal Health and Epidemiology Center, Qingdao, 266032 China; 3 Key Laboratory of Animal Biosafety Risk Prevention and Control (South), Ministry of Agriculture and Rural Affairs, Qingdao, China

**Keywords:** ASFV, repeat sequences, evolution, genetic diversity

## Abstract

African swine fever virus (ASFV) is a large DNA virus that infects domestic pigs with high morbidity and mortality rates. Repeat sequences, which are DNA sequence elements that are repeated more than twice in the genome, play an important role in the ASFV genome. The majority of repeat sequences, however, have not been identified and characterized in a systematic manner. In this study, three types of repeat sequences, including microsatellites, minisatellites and short interspersed nuclear elements (SINEs), were identified in the ASFV genome, and their distribution, structure, function, and evolutionary history were investigated. Most repeat sequences were observed in noncoding regions and at the 5’ end of the genome. Noncoding repeat sequences tended to form enhancers, whereas coding repeat sequences had a lower ratio of alpha-helix and beta-sheet and a higher ratio of loop structure and surface amino acids than nonrepeat sequences. In addition, the repeat sequences tended to encode penetrating and antimicrobial peptides. Further analysis of the evolution of repeat sequences revealed that the pan-repeat sequences presented an open state, showing the diversity of repeat sequences. Finally, CpG islands were observed to be negatively correlated with repeat sequence occurrences, suggesting that they may affect the generation of repeat sequences. Overall, this study emphasizes the importance of repeat sequences in ASFVs, and these results can aid in understanding the virus's function and evolution.

**Supplementary Information:**

The online version contains supplementary material available at 10.1186/s13567-022-01119-9.

## Introduction

African swine fever (ASF) is a haemorrhagic and devastating infectious disease of pigs caused by the African swine fever virus (ASFV), with a mortality rate of up to 100% [1, 2]. ASFV has a significant impact on the pig industry in many countries [3]. Over fifty countries in Africa, Europe, and Asia have experienced ASF outbreaks [3, 4]. ASF first appeared in China in August 2018 and spread quickly throughout nearly all of the country's provinces [5]. The viral genome is double-stranded DNA, with sizes ranging from 170 to 194 kb [6]. The ASFV genome encodes more than 150 open reading frames (ORFs), which form a mature ASFV virion with a large, enveloped, and complicated architecture, making the development of an effective vaccine and drug difficult due to an incomplete understanding of the virus [6–8].

Repeat sequences are DNA sequences that appear repeatedly in the genome and are crucial for viral infection and variation [9–11]. According to the current findings, repeat sequences are widely distributed in ASFV genomes and may promote the occurrence of homologous recombination [12]. A few repeat sequences have been found to play an important role in ASFV infection in host cells [11, 13]. For example, the repeat peptide ([KPCPPP]_3_) acts as a cell-penetrating peptide, potentially assisting the virus in entering the cell [11]. In addition, variation in the number of repeat sequences within genes or intergenic regions contributes to the genetic diversity of ASFV [12, 14, 15]. Although the functions of a small number of repeat sequences in the virus have been identified, the structure, function, and evolution of the vast majority of repeat sequences have not been studied [13, 15]. A systematic study of ASFV repeat sequences is required to bridge this gap, which will aid in virus prevention and control.

Tandem repeats and SINEs are the most common types of repeat sequences [16, 17]. They not only have an impact on evolution, inheritance, and variation but also play an important role in gene expression, transcription, and regulation [18, 19]. For example, tandem repeats at the ends of human chromosomes play an important regulatory role in cell growth, proliferation, and apoptosis processes, such as maintaining chromosome integrity and stability, preventing chromosome degradation by nucleases, avoiding chromosome end fusion and recombination, providing telomerase substrates, and regulating cell lifecycles [20, 21]. SINEs are found in many vertebrate and invertebrate species and have lineage specificity, which plays a particularly important role in the regulation of gene expression and the creation of RNA genes [22, 23]. For example, SINEs containing CTCF binding sites can be used as boundary elements to influence chromatin structure and transcription [22, 24]. In this study, the ASFV repeat sequences were systematically identified and classified, and their structure, function, and evolutionary characteristics were further investigated. The findings demonstrated that repeat sequences are essential for ASFV expression and evolution. This work will contribute to a better understanding of ASFV function and evolution and pave the way for future research on repeat sequences in other viruses.

## Materials and methods

### Identification of repeat sequences in ASFV genomes

A total of 86 ASFV genome sequences were obtained from the NCBI GenBank [25] database on August 15, 2021. The gaps and nonstandard bases were removed from the ASFV genomes. REPuter (version 1) [26] and TRF (version 1) [27] were used to identify repeat sequences in ASFV genomes with the default parameters.

### Annotation of ASFV genomes

Genes encoded in ASFV genomes and their relevant proteins were predicted using GeneMarkS (version 4.28) [28] with the default parameters. To infer the function of the ASFV proteins, they were queried against all ASFV proteins downloaded from the NCBI RefSeq and ASFVdb [29] databases using the NCBI BLAST program (version 2.9.1) [30].

### Structure‒function analysis of repeat sequences in ASFV genomes

As shown in Table 1, the structure and function of repeat sequences were investigated using a variety of publicly available tools.

**Table 1:** **Software tools for predicting the structure and function of repeat sequences**

Tool name	Function
iEnhancer-EL [31]	Identifying enhancers with the ensemble learning approach
iPromoter-2L [32]	A two-layer predictor for identifying promoters by multiwindow-based K-tuple nucleotide composition
Espritz [33]	Detecting disordered regions from primary sequences by extracting the relevant information from the local context of the residue under consideration using the bidirectional recursive neural network
NetSurfP-2.0 [34]	Predicting the secondary structure for each residue of the input sequences by using an architecture composed of convolutional and long short-term memory neural networks
CAMP_R3_ [35]	Multiple machine learning algorithms for predicting antimicrobial peptides based on the amino acid sequence
MLCPP [36]	Machine-learning-based prediction of cell-penetrating peptides

### Inference of phylogenetic trees and determination of genotypes

Previously published research identified 24 genotypes based on the C-terminus of the p72 gene [37]. To determine the genotype of all analysed ASFV strains, we used MAFFT (version 7) [38] to align the genomic sequences in the 415-bp C-terminal region of the p72 gene; then, we inferred phylogenies using the maximum-likelihood algorithm in MEGAX [39] with 100 bootstrap replicates. The genotype of each ASFV was assigned based on previous studies [37, 40].

### Pan-genomic analysis of the ASFV repeat sequence

Repeat sequences were clustered by treating identical repeat sequences as a cluster. The characteristic curves of the ASFV pan-repeat sequences and the core-repeat sequences were portrayed using PanGP with DG sampling algorithms [41]. The flower plot depicting the number of core and dispensable repeat sequence clusters, including unique repeat sequences in ASFV, was generated by the *plotrix()* function in R (version 4.0.3) [42].

### Identification of CpG islands

The CpG islands in the ASFV genomes were detected using Cpgplot (version 1) [43].

### Statistical analysis

All statistical analyses were conducted in R (version 4.0.3) [42] and Python (version 3.6) [44]. The Wilcoxon rank-sum test was conducted using the *wilcox.test()* function in R. The correlation coefficient was calculated using the *cor.test()* function in R.

## Results

### Identification, classification and distribution of repeat sequences in ASFV genomes

**Figure 1:**
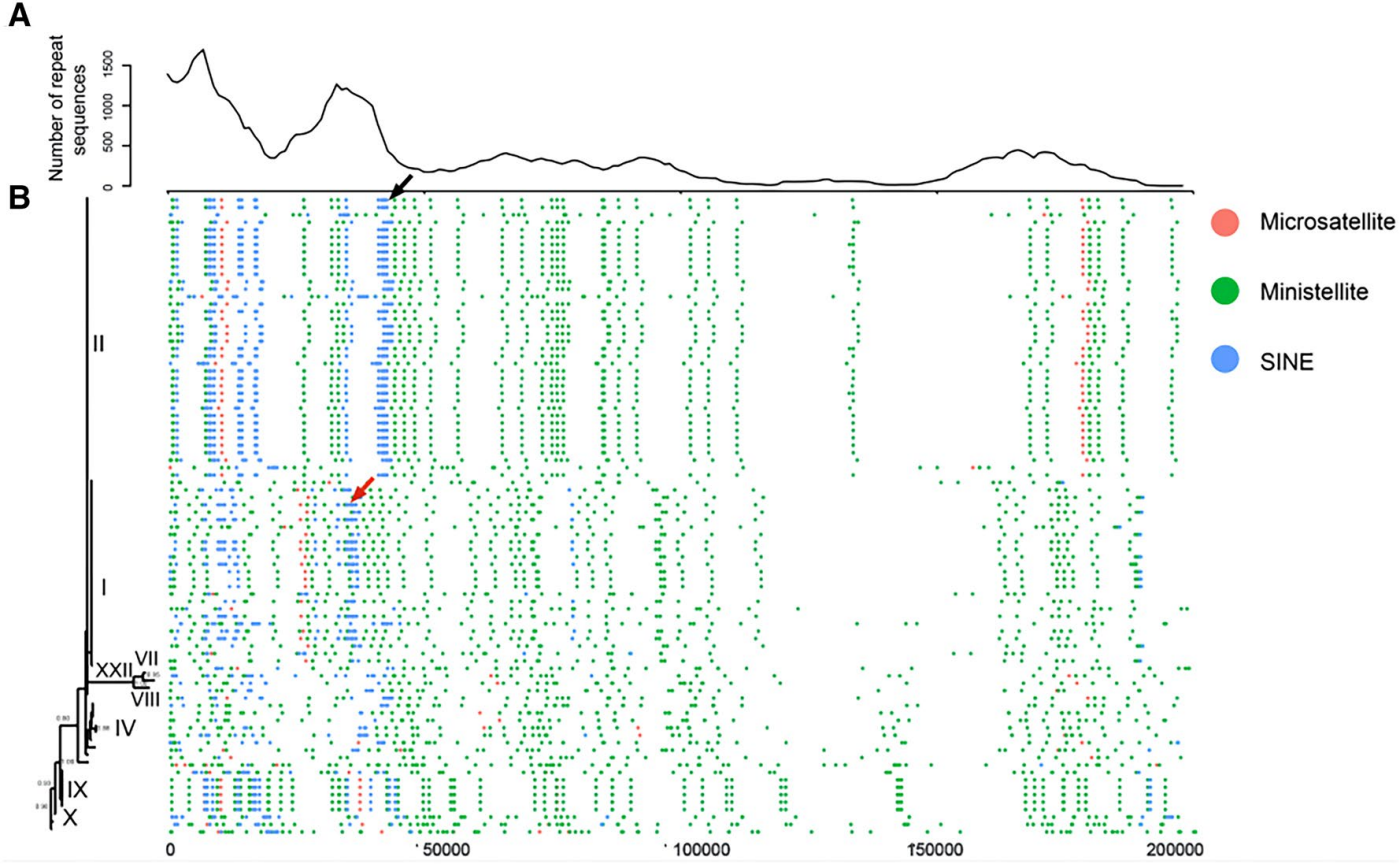
**The number, position, and distribution of repeat sequences in the genome.** **A** The number of repeat sequences identified in 86 genomes (per kb). **B** The phylogenetic tree on the left side was built with MEGA based on the C-terminal sequences (415 bp) of the p72 gene (see Materials and methods), and the genotypes of ASFV isolates are labelled with Greek numerals. The three types of repeat sequences in ASFV genomes are indicated by different colours. The black and red arrows refer to examples of genotype-specific repeat sequences in genotypes II and I, respectively.

A total of 86 genome sequences of ASFVs were obtained from the NCBI GenBank database; these are listed in Additional file 1. The ASFVs were found to contain an abundance of repeat sequences (Figure 1). These repeat sequences were divided into three types: (1) microsatellites, which are defined as small tandem repeat sequences with less than ten bp in the repeat unit, (2) minisatellites, which are defined as small tandem repeat sequences with 10–300 bp in the repeat unit, and (3) short interspersed nuclear elements (SINEs), which are defined as a discontinuous distribution of repeat units of 100–500 bp. The analysis of the location of repeat sequences in ASFV genomes revealed that the bulk of repeat sequences were located in either the 5′ or 3′ ends of genomes (Figure 1A). Specifically, more than 90% of microsatellites and SINEs were located at the 5′ end of the ASFV genome, while minisatellites were irregularly distributed throughout the genome.

The vast majority of the repeat sequences were found to be genotype specific. The retrieved genomes contained nine genotypes. Most of the ASFV genomes, including those recently found in Europe and China, were genotype I or II. Genotype I included 26 ASFV genomes, with more than 30% of repeat sequences being genotype specific (see examples marked by the red arrow in Figure 1B). Genotype II encompassed 38 ASFV genomes, with more than half of the repeat sequences being genotype specific (see examples marked by the black arrow in Figure 1B).

**Figure 2:**
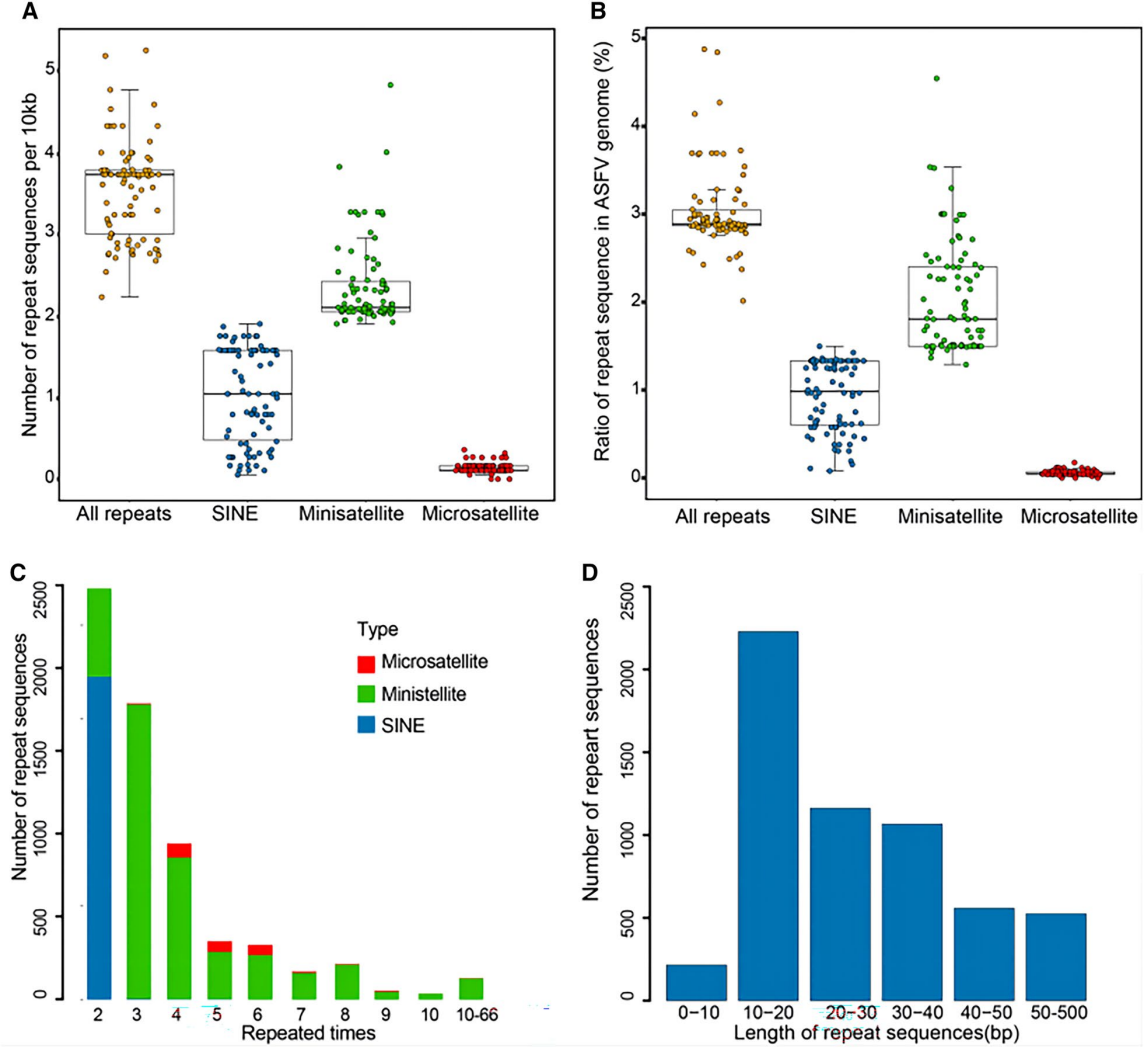
**The distribution of three types of repeat sequences in the ASFV genome**. **A** The number of different types of repeat sequences per 10 kb in 86 ASFV genomes. **B** The ratio of genomic regions covered by three types of repeat sequences in 86 ASFV genomes. **C** The number of repeat units for the three repeat sequence types in 86 ASFV genomes. **D** The number of repeat units of different lengths. The microsatellites, minisatellites, and SINEs are coloured red, green, and blue, respectively.

The number of repeat sequences and ratio of repeat sequences in 86 ASFV genomes were analysed, and the ASFVs were identified to have 2.2 to 5.3 repeat sequences per 10 kb of genomic sequence, with a median of 3.7 (Figure 2A). More than 60% of these repeat sequences were minisatellites, with the number ranging from 1.9 to 4.9 per 10 kb of genomic sequence. The minisatellites covered a median of 1.8% of the ASFV genomes (Figure 2B). Approximately 30% of repeat sequences were made up of SINEs, with numbers ranging from 0.05 to 1.9 per 10 kb of genomic sequence, covering a median of 0.9% of the ASFV genomes. The remaining repeat sequences belonged to microsatellites, which had fewer than 0.5 per 10 kb of genomic sequence.

As the repeat units served as the basic units of repeat sequences, the number of repeat units was analysed in ASFV genomes (Figure 2C). The number of repeat units ranged from 2 to 66, with a median of 3. In approximately 80% of the repeat sequences, the number of repeat units was less than four times; in only 2% of the repeat sequences, the number of repeat units was more than ten times. The repeat unit was only repeated two times for nearly all SINEs and three times for more than 40% of minisatellites.

The length of repeat units in repeat sequences was then analysed (Figure 2D). The repeat unit lengths varied from 1 to 281 bp, with a median of 24 bp. Among them, more than half of the repeat units were no longer than 30 bp, and approximately 9% of the repeat units were more than 50 bp. Interestingly, the most common repeat unit length was 10–20 bp.

### A high incidence of repeat sequences in noncoding regions

**Figure 3:**
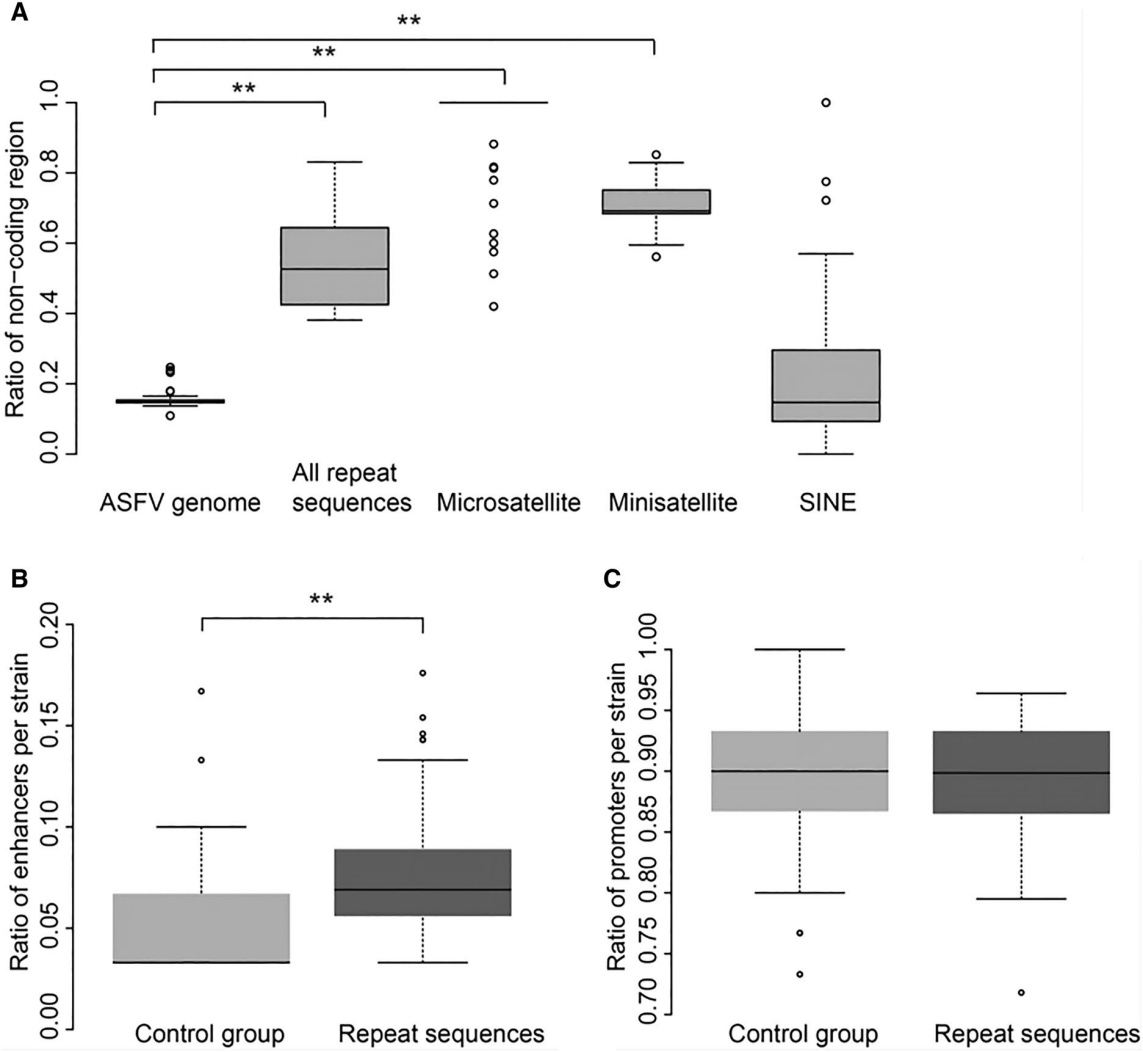
**The function of repeat sequences in noncoding regions.** **A** The distribution of the noncoding ratio for three types of repeat sequences in the ASFV genome. **B** Comparison of the ratio of enhancers in the repeat and nonrepeat sequences in ASFV genomes. **C** Comparison of the ratio of promoters in repeat and nonrepeat sequences in ASFV genomes. “**”, *p* value < 0.01.

We evaluated the ratios of repeat sequences in the noncoding and coding regions and discovered that approximately 50% of repeat sequences were located in noncoding regions (Figure 3A). Specifically, approximately 70% of minisatellites and 95% of microsatellites were located in noncoding regions, while less than 20% of SINEs were located in noncoding regions. The analysis of the SINEs in coding regions revealed that 70% of them were located in the multigene family (MGF) proteins, with the remaining SINEs found in other proteins and unknown proteins (Additional file 2). This indicated that SINEs were likely to cause amino acid insertions or deletions, thereby influencing the functions of the ASFV proteins.

### Characterization of the structure and function of repeat sequences in noncoding regions

The role of repeat sequences in transcription regulation was investigated because most repeat sequences were located in noncoding regions (Figure 3A). In the repeat sequences located in noncoding regions, two kinds of functional elements were identified: enhancers and promoters. For comparison, the same number of nonrepeat sequences with the same size as repeat sequences (control group) were randomly selected from the noncoding regions in each ASFV genome. The ratio of enhancers in repeat sequences was significantly higher than that in nonrepeat sequences (0.08 vs. 0.04, *p* value < 0.01) (Figure 3B). Surprisingly, the median ratio of promoters in both repeat sequences and the control group was greater than 0.9 (Figure 3C), and no significant difference was observed between them.

### Characterization of the sequence, structure and function of repeat sequences in coding regions

**Figure 4:**
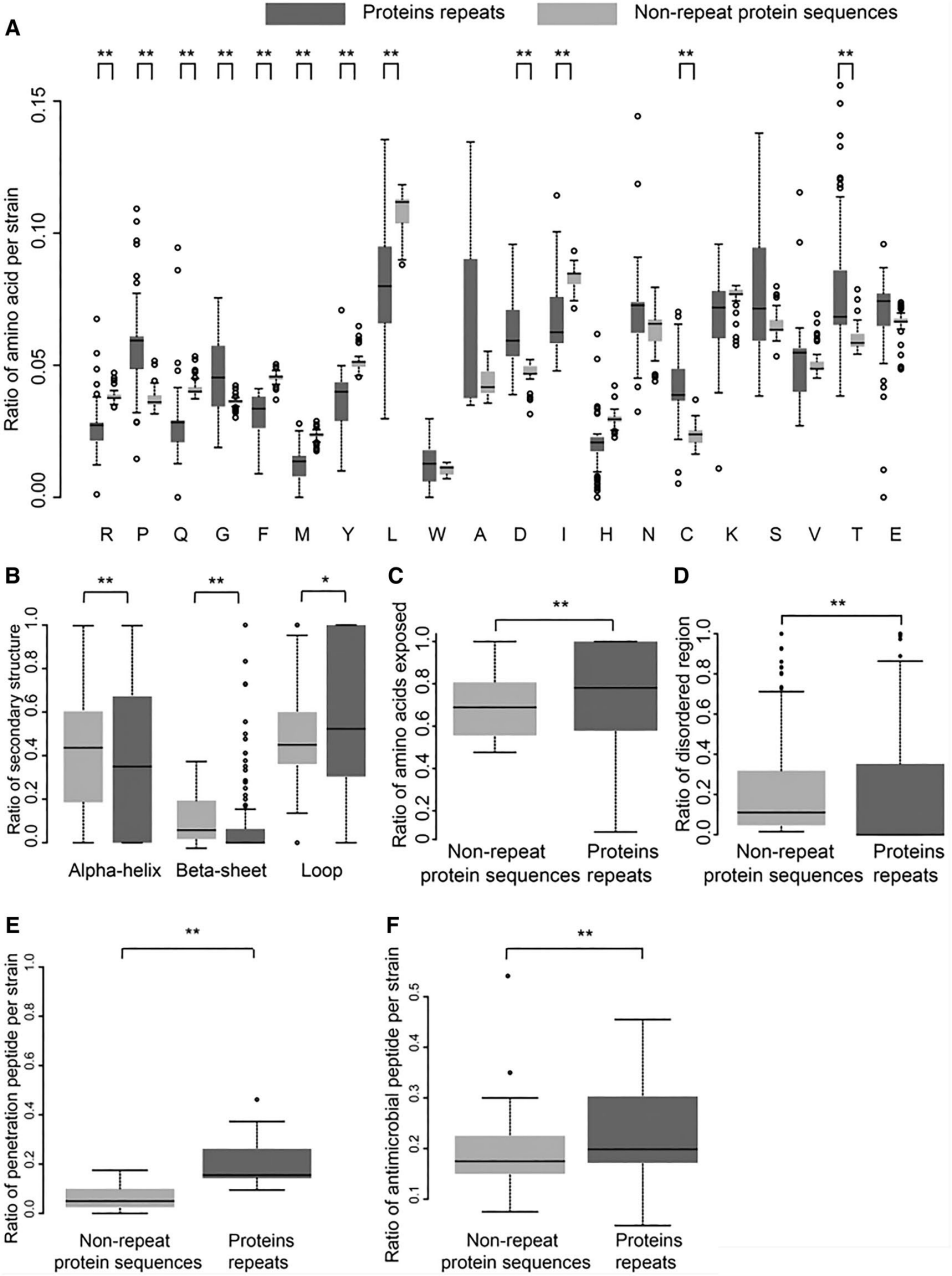
**The characteristics of repeat sequences in coding regions**. **A** The ratio of amino acids in repeat protein sequences versus nonrepeat protein sequences in ASFV strains. **B** The ratio of secondary structures in repeat protein sequences versus nonrepeat protein sequences. **C** The ratio of amino acids exposed in repeat protein sequences versus nonrepeat protein sequences. An exposed amino acid was defined as a residue with a relative solvent accessible surface area (rASA) greater than 0.25. **D**–**F** The ratio of disordered, penetrating peptides and antimicrobial peptides in repeat protein sequences versus nonrepeat protein sequences. For subfigures B to D, only nonredundant protein sequences were used. “*”, *p* value < 0.05; “**”, *p* value < 0.01.

Then, the repeat sequences in coding regions were analysed. For each repeat sequence, the protein sequence encoded by the repeat sequence (defined as repeat protein sequences for clarity) and other protein sequences in the same protein (defined as nonrepeat protein sequences) were used in the analysis. The amino acid ratios in repeat protein sequences were calculated and compared to those in the nonrepeat protein sequences. Interestingly, large differences in amino acid ratios were observed in the two types of protein sequences (Figure 4A). The ratios of several hydrophobic amino acids, including phenylalanine (F), methionine (M), leucine (L), and isoleucine (I), were significantly lower in the repeat protein sequences than in the nonrepeat protein sequences, while the ratios of three hydrophilic amino acids, including cysteine (C) and threonine (T), were significantly higher in the repeat protein sequences than in the nonrepeat protein sequences (*p* values < 0.01).

We further investigated the structural characteristics of the repeat protein sequences. The ratios of three kinds of secondary structure elements were calculated (alpha-helix, beta-sheet, and loop). The median ratio of alpha-helix in the repeat protein sequences was 0.35, which was significantly lower than that in nonrepeat protein sequences (0.35 vs. 0.43, *p* value < 0.01) (Figure 4B), and the median ratio of beta-sheet in the repeat protein sequences was only 0.0, which was significantly lower than that in nonrepeat protein sequences (0.0 vs. 0.06, *p* value < 0.01). Interestingly, the median ratio of loops in the repeat protein sequences was 0.52, which was significantly higher than that in nonrepeat protein sequences (0.52 vs. 0.45, *p* value < 0.05). Then, the ratio of amino acids exposed was calculated, and the repeat protein sequences were observed to have a higher ratio of amino acids exposed than the nonrepeat protein sequences (Figure 4C). In addition, the ratio of amino acids in the disordered region, which is defined as the region unlikely to form a defined protein three-dimensional structure, was calculated, and the repeat protein sequences were observed to have a lower ratio of amino acids in the disordered region than the nonrepeat protein sequences (Figure 4D).

The potential functions of the repeat protein sequences were further analysed. Previous studies have shown that repeat protein sequences can act as penetrating peptides or antimicrobial peptides in herpes simplex and human respiratory syncytial viruses [11, 45]. Thus, penetrating and antimicrobial peptides were identified in repeat protein sequences. A median of 18% and 21% of repeat protein sequences were observed to have the function of penetrating peptides and antimicrobial peptides, respectively (Figures 4E and F), which were significantly higher than those in nonrepeat protein sequences. Taken together, more than 30% of the repeat protein sequences functioned as antibacterial peptides or penetrating peptides (Additional file 3), suggesting that the repeat protein sequences may play an important role in ASFV infection.

### Pan-genomic analysis of repeat sequences in the ASFV genome

**Figure 5:**
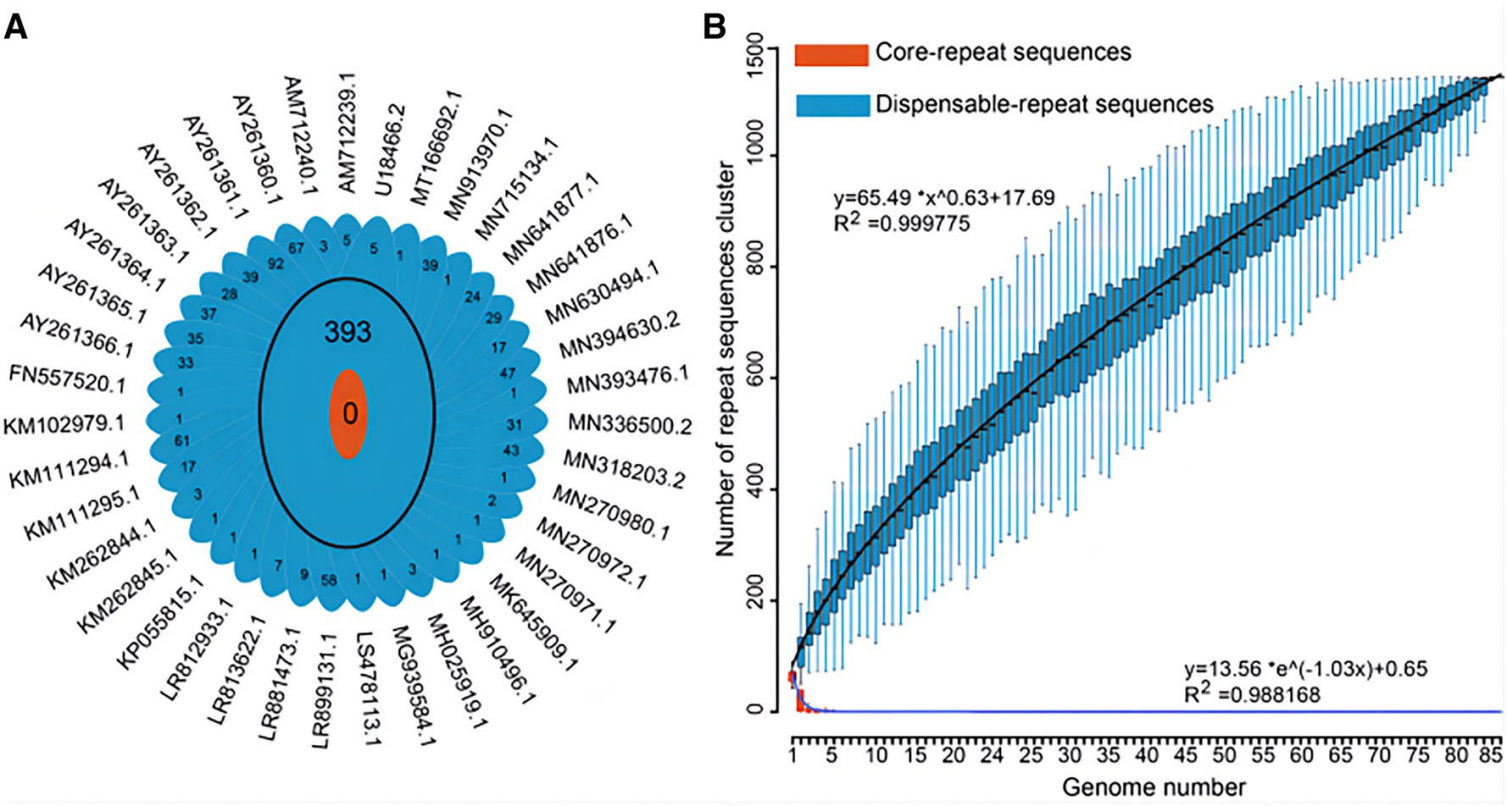
**Pan-repeat sequence analysis of ASFVs.** **A** Flower plot showing the core repeat and dispensable repeat sequence clusters of the 86 ASFV strains. The diagram depicts the core repeat sequence cluster number (orange) and the dispensable repeat sequence cluster number (blue) for the 86 ASFV strains. The number of unique repeat sequences in each strain is shown beside the accession number of the ASFV genomes. For clarity, only the strains with unique repeat sequences are shown. **B** The relationship between genome number and dispensable repeat and core repeat sequence profiles. The black and blue curves refer to the least-squares fitting for the average number of dispensable repeat clusters and core repeat clusters versus the number of genomes, respectively. The mathematical functions and the R-squared values of both models are delineated on the graph.

The pan-genomic analysis was conducted for the repeat sequences in ASFV genomes. All the repeat sequences identified in the ASFV genomes were defined as pan-repeat sequences. They were classified into 1141 clusters based on sequence similarity at the 100% level. Two kinds of clusters were obtained: core repeat clusters, which appeared in all ASFV genomes, and dispensable repeat clusters, which appeared in one or more ASFV genomes. Among the dispensable repeat clusters, those that only appeared in one genome were called unique repeat sequences. The analysis of pan-repeat sequences showed that no core repeat clusters were found, despite the presence of 1141 dispensable repeat clusters, including 748 unique repeat sequences (Figure 5A).

With the increase in the number of genomes, the number of dispensable repeat clusters was fitted by the power law regression model (y_pan_ = A_pan*_x^Bpan^ + C_pan_), while the number of core repeat clusters was fitted by the exponential model (y_core_ = A_core*_e^Bcore*x^ + C_core_), according to Zhao’s study [41]. The mathematical functions of both models were delineated on the graph. The B_pan_ in the power law regression model was greater than 0.5, suggesting that the pan-repeat sequences of ASFVs are in an open state (see the black curve in Figure 5B). This trend reflects that ASFVs have flexible repeat sequences, and the size of the pan-repeat may expand with each added genome, which contributes to new repeat sequences. As the number of analysed genomes increased, the number of core repeat sequence clusters presented a significant decrease and became zero (see the blue curve in Figure 5B). This trend reflects the diversity of repeat sequences in the ASFV genome.

Considering the genotype specificity of repeat sequences, we further analysed the pan-repeat sequences of genotypes I and II. Overall, 5 and 32 core repeat clusters and 251 and 73 dispensable repeat clusters, including 138 and 22 unique repeat sequences, respectively, were observed, which constituted the ASFV genotype I and II pan-repeats (Additional file 4). The B_pan_ in the power law regression model of genotypes I and II was greater than 0.5 (Additional file 4), suggesting that the pan-repeat sequences within the genotype are also in an open state. As the number of analysed genomes increased, the core-repeat cluster curve presented a converging trend. This trend reflects that repeat sequences within genotypes are conserved during evolution.

### The relationship between CpG islands and repeat sequences in ASFV genomes

**Figure 6:**
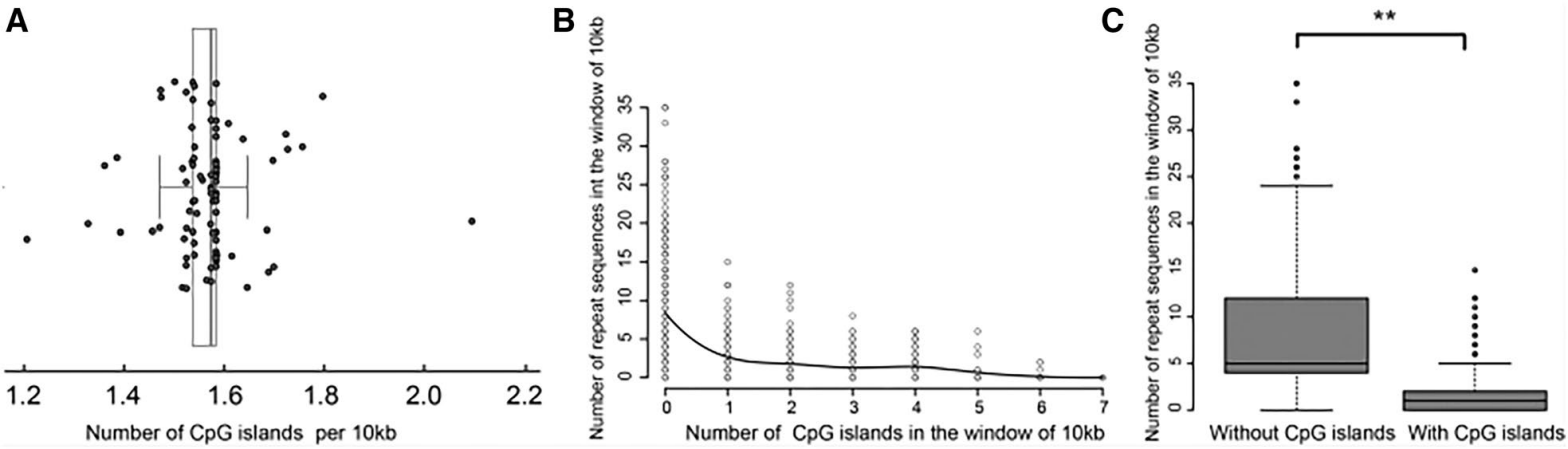
**Distribution of the CpG islands and repeat sequences in each genome.** **A** The number of CpG islands per 10 kb in each ASFV genome. **B** The number of repeat sequences in a sliding window of 10 kb (with a step size of 1 kb) versus the number of CpG islands. **C** Comparison of the number of CpG islands in a sliding window of 10 kb (with a step size of 1 kb) with and without CpG islands in ASFV genomes. “**”, *p* value < 0.01.

Previous studies have shown that CpG islands help stabilize the genome and may prevent the formation of repeat sequences. Thus, the influence of CpG islands on repeat sequences was investigated. The number of CpG islands identified in ASFV genomes ranged from 1.2 to 2.1 per 10 kb of genomic sequence (Figure 6A), with a median of 1.57. Interestingly, the number of CpG islands identified per 10 kb window of ASFV genomes was observed to have a negative correlation with the number of repeat sequences identified in the same region (Pearson correlation coefficient = −0.48) (Figure 6B). Specifically, the windows with CpG islands had a median of only one repeat sequence, while those without CpG islands had a median of five repeat sequences (Figure 6C).

## Discussion

The repeat sequences in the ASFV genome were studied for their classification, distribution, structure, function, and evolution. In this study, four features of ASFV repeat sequences were revealed for the first time. First, the repeat sequences were enriched at the 5’ end of the ASFV genome. This is consistent with Campbell et al.’s study that discovered a large number of simple sequence repeats (SSRs) within the terminal region of the Fowlpox virus genome [46]. The repeat sequences tended to be located in noncoding regions because insertions and deletions of repeat sequences in the coding region may disrupt the ORF, which may cause severe damage or even death to the virus [47, 48]. Therefore, only a few repeat sequences were observed in the coding regions of viral genomes such as Dengue virus (DEN) and Yellow fever [49, 50]. However, repeat sequences in noncoding regions also play an important role in the viral life cycle [51–53]. Some repeat sequences can affect the replication and transcription of viruses [51, 53]. For example, the repeat sequences of the herpes simplex virus contain a promoter of a gene that can regulate virus gene transcription [51]. Thus, our results provide evidence to conclude that repeat sequences are structural elements of the ASFV genome that underpin its distinct functionality.

Second, repeat sequences were predicted to have a higher ratio of loop structures and a lower ratio of disorder regions compared to nonrepeat protein sequences. Similar to the results of previous studies, repeat protein sequences had a large ratio of loop structures and could provide more protein binding sites [54, 55]. The repeat sequences may be less likely to form disordered regions due to their flexible nature. Because the repeat sequences contain a low ratio of polar amino acids (such as tyrosine (Y) and arginine (R)), it is difficult to form polyelectrolytes, which are an important part of the disordered region [56]. Based on these results, we conclude that repeat sequences are important in the conformation of ASFV-specific structural peptides.

Third, our results demonstrated that the ASFV repeat sequences tended to encode penetrating peptides and antimicrobial peptides. A small number of repeat sequences have been identified to encode penetrating peptides and antimicrobial peptides [45, 57]. The repeat sequences encoding penetrating peptides can play an important role in ASFV entering host cells by receptor-mediated endocytosis [11]. Moreover, the repeat sequences encoding antimicrobial peptides can help in the development of new antimicrobial peptide drugs [58, 59], as antimicrobial peptides originating from viral proteins are key players in the development of innovative drug delivery systems [45, 57]. These findings imply that the ASFV repeat sequences have a specific role in functional peptides.

Fourth, our results showed that the pan-repeat sequences of ASFV presented an open state and that core repeats were non-existent in all of the analysed strains. Similar to bacteria, open pan-repeat sequences indicate that the genetic material of ASFV may be constantly mutated [60]. The core repeat sequences in ASFV were not observed, which suggests that ASFV repeat sequences are diverse. Furthermore, repeat sequences have been reported to be related to homologous recombination [12]. As a result, the diversity of repeat sequences can further promote the diversity of ASFV. Overall, our findings underscore the involvement of repeat sequences in the genomic evolution and variation of ASFV, highlighting the importance of further investigation into repeat sequences in other viruses.

This study has two limitations that need to be acknowledged. First, the location, size and number of repeat sequences observed in ASFV genomes may be affected by the repeat sequence identification algorithm. For comprehensive detection of repeat sequences, two complementary methods (TRF and REPuter) were used for identifying repeat sequences in ASFV genomes. Second, the predicted structure and functional features of repeat sequences have not been examined experimentally. More efforts are needed to validate these predictions and to clarify the roles of repeat sequences of the ASFV genome.

In summary, this study is the first to illustrate the distribution, structural, functional, and evolutionary properties of repeat sequences in ASFVs, thus advancing our understanding of the virus and facilitating the development of effective anti-ASFV medications. The methodological framework established in this study can be utilized to guide future research on repeat sequences of other viruses.

## Supplementary Information

**Additional file 1. The viruses used in this study.**

**Additional file 2. The functional classification of ASFV proteins with short interspersed repeated sequence (SINE).**

**Additional file 3. The intersection of penetrating peptides and antimicrobial peptides in repeat protein sequences.**

**Additional file 4. Pan-repeat sequence analysis of ASFV genotypes I and II.** (A and C) Flower plot showing the core repeat and dispensable repeat sequence clusters of ASFV genotypes I and II, respectively. The diagram depicts the core repeat sequence cluster number (orange) and the dispensable repeat sequence cluster number (blue) for ASFV genotypes I and II, respectively. The number of unique repeat sequences in each strain is shown beside the accession number of the ASFV genome. (B and D) The relationship between genome number and dispensable repeat and core repeat sequence profiles for ASFV genotypes I and II, respectively. The black and blue curves refer to the least-squares fitting for the average number of dispensable repeat clusters and core repeat clusters versus the number of genomes, respectively. The mathematical functions and the R-squared values of both models are delineated on the graph.
