# Supplementary material for: Systematic identification and characterization of repeat sequences in African swine fever virus genomes
Source: Vet Res. 2022 Dec 2;53:101. doi: 10.1186/s13567-022-01119-9 (PMC9717548; doi:10.1186/s13567-022-01119-9)
Supplement: Supplementary file 1 — Additional file 1. The viruses used in this study. [file 13567_2022_1119_MOESM1_ESM.doc]

Accession number	Size (bp)	Number of CDS	Accession number	Size (bp)	Number of CDS	
AY261360.1	193886	152	MH025918.1	188630	147	
AY261362.1	192714	147	MH025920.1	188629	147	
LR881473.1	192206	146	MH025917.1	188628	148	
MN194591.1	191911	183	MH025916.1	188627	147	
KM111294.1	191058	153	MH025919.1	188611	147	
AY261365.1	190773	149	MN641877.1	188502	141	
MN715134.1	190601	147	MN913970.1	188277	144	
LR536725.1	190599	147	MN336500.2	187866	163	
LR722599.1	190598	147	AY261361.1	187612	145	
MT748042.1	190597	147	AY261366.1	186528	147	
LR722600.1	190594	147	MT180393.1	186498	147	
LR899193.1	190592	148	MN394630.2	186119	169	
FR682468.2	190584	147	AY261364.1	185689	143	
MN393476.1	190576	147	MN630494.1	185338	145	
AY261363.1	190324	148	MN641876.1	185293	140	
MK543947.1	190202	147	KX354450.1	184638	141	
LR812933.1	190145	148	MN270977.1	184581	142	
LR899131.1	189950	153	KM111295.1	184368	145	
MH910495.1	189465	152	MN270979.1	184206	140	
MT847622.1	189422	148	MN270975.1	183724	140	
MT847620.1	189414	148	MN270972.1	183723	140	
MT847621.1	189409	148	MN270974.1	183720	139	
MG939585.1	189405	148	MN270971.1	183645	139	
MG939587.1	189405	147	MN270970.1	183639	138	
MN172368.1	189405	148	MN270969.1	183636	139	
MK333180.1	189404	148	MN318203.2	183291	169	
MK940252.1	189403	148	LR813622.1	183186	144	
MG939583.1	189401	148	KM102979.1	182906	139	
MG939588.1	189401	148	LS478113.1	182446	136	
MG939584.1	189399	148	KM262844.1	182362	138	
MK628478.1	189399	148	AM712239.1	182284	140	
MH681419.1	189394	148	MN270980.1	181869	140	
MK645909.1	189394	148	MN270973.1	181816	140	
MG939586.1	189393	147	MT932579.1	181761	141	
MG939589.1	189393	149	MT932578.1	181759	141	
MK128995.1	189393	148	MN270978.1	181699	141	
MT496893.1	189393	148	MN270976.1	181651	143	
KJ747406.1	189387	148	FN557520.1	181187	146	
MT847623.1	189356	148	KP055815.1	180365	135	
MH766894.1	189354	145	KM262845.1	172051	134	
KP843857.1	189333	149	AM712240.1	171719	134	
MH910496.1	189315	148	U18466.2	170101	125	
MT459800.1	189252	147	MT166692.1	166931	129	
