# Supplementary figures and images for: Systematic identification and characterization of repeat sequences in African swine fever virus genomes
Source: Vet Res. 2022 Dec 2;53:101. doi: 10.1186/s13567-022-01119-9 (PMC9717548; doi:10.1186/s13567-022-01119-9)

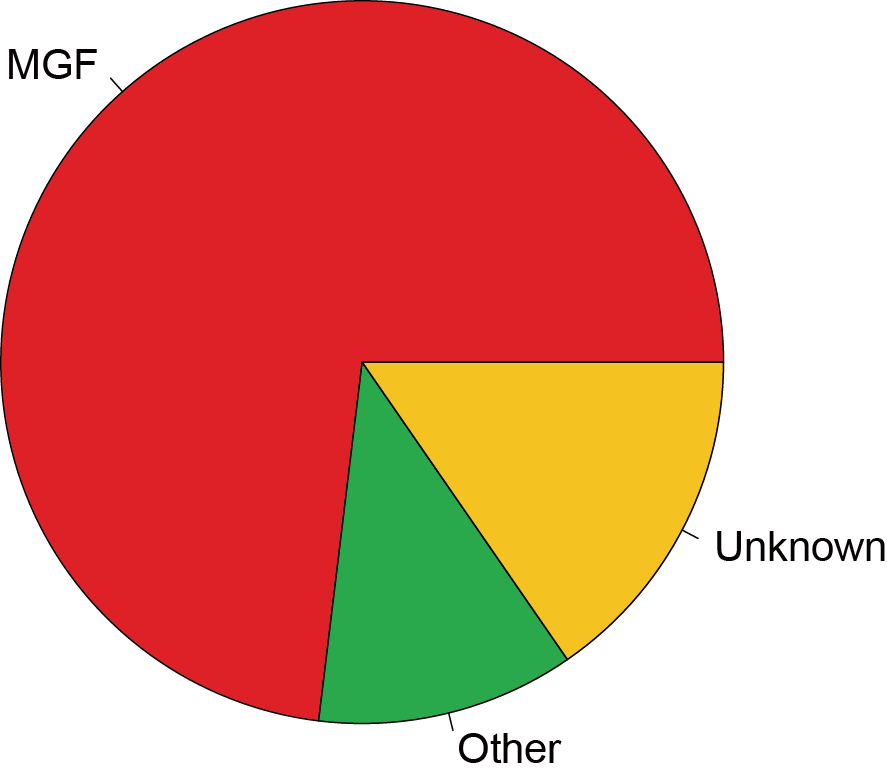

Supplement: Supplementary file 2 — Additional file 2. The functional classification of ASFV proteins with short interspersed repeated sequence (SINE). [file 13567_2022_1119_MOESM2_ESM.tif]

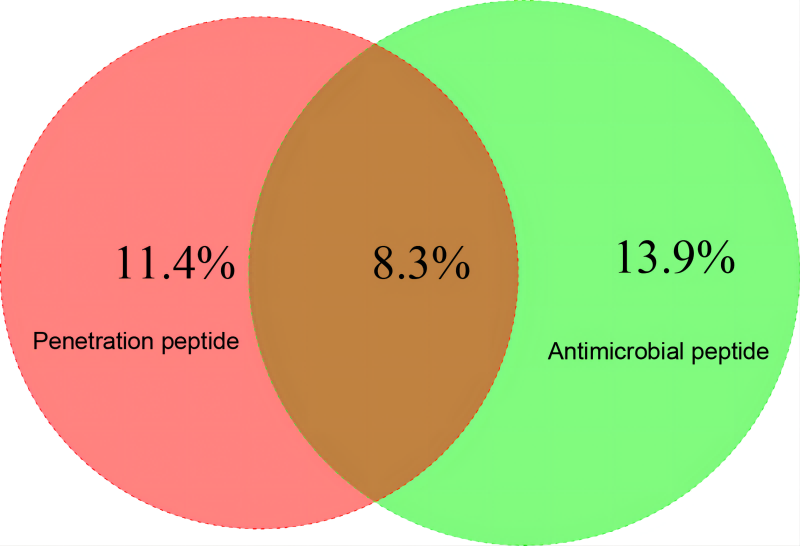

Supplement: Supplementary file 3 — Additional file 3. The intersection of penetrating peptides and antimicrobial peptides in repeat protein sequences. [file 13567_2022_1119_MOESM3_ESM.tif]

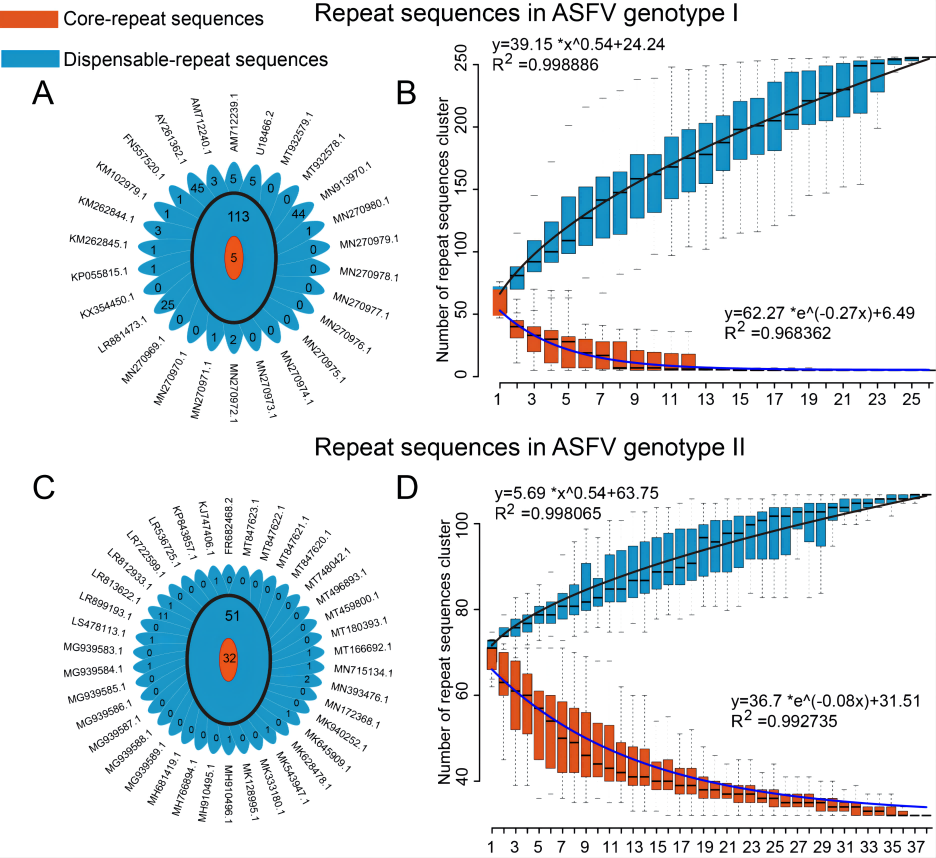

Supplement: Supplementary file 4 — Additional file 4. Pan-repeat sequence analysis of ASFV genotypes I and II. (A and C) Flower plot showing the core repeat and dispensable repeat sequence clusters of ASFV genotypes I and II, respectively. The diagram depicts the core repeat sequence cluster number (orange) and the dispensable repeat sequence cluster number (blue) for ASFV genotypes I and II, respectively. The number of unique repeat sequences in each strain is shown beside the accession number of the ASFV genome. (B and D) The relationship between genome number and dispensable repeat and core repeat sequence profiles for ASFV genotypes I and II, respectively. The black and blue curves refer to the least-squares fitting for the average number of dispensable repeat clusters and core repeat clusters versus the number of genomes, respectively. The mathematical functions and the R-squared values of both models are delineated on the graph. [file 13567_2022_1119_MOESM4_ESM.tif]
